# Literature review of the clinical characteristics of metformin-induced hepatotoxicity

**DOI:** 10.3389/fphar.2022.969505

**Published:** 2022-09-06

**Authors:** Chunjiang Wang, Hongyi Deng, Yunfei Xu, Ying Liu

**Affiliations:** ^1^ Department of Pathophysiology, Xiangya School of Medicine, Central South University, Changsha, Hunan, China; ^2^ Sepsis Translational Medicine Key Lab of Hunan Province, Changsha, Hunan, China; ^3^ Department of Pharmacy, The Third Xiangya Hospital, Central South University, Changsha, Hunan, China; ^4^ Department of Pharmacy, Hunan Provincial Maternal and Child Health Care Hospital, Changsha, Hunan, China

**Keywords:** metformin, cholestasis, hepatotoxicity, hepatocellular, liver injury

## Abstract

**Background:** Knowledge of metformin-induced hepatotoxicity is based on case reports. The aim of this study was to investigate the clinical features of metformin-induced hepatotoxicity.

**Methods:** We collected relevant literature on metformin-induced hepatotoxicity published from January 1994 to February 2022 by searching Chinese and English databases.

**Results:** Thirty patients (19 males and 11 females) from 29 articles were included, with a median age of 61 years (range 29–83). The median time to onset of liver injury was 4 weeks (range 0.3–648) after metformin administration. Clinical symptoms occurred in 28 patients, including gastrointestinal reactions (56.7%), jaundice (50.0%), fatigue (36.7%), anorexia (23.3%), pruritus (13.3%), dark urine (13.3%), and clay-colored stools (10.0%). Serum alanine transaminase, aspartate transaminase, γ-glutamyl transferase, total bilirubin and alkaline phosphatase were elevated to varying degrees. Liver imaging in 26 patients showed hepatic steatosis (6 cases, 23.1%) and gallbladder wall thickening (11.5%). Liver biopsies from 13 patients showed portal phlebitis (61.5%), cholestatic hepatitis (38.5%), and parenchymal inflammation (38.5%). After metformin discontinuation, liver function returned to normal levels at a median of 6 weeks (range 2–16).

**Conclusions:** Metformin-induced hepatotoxicity is a rare adverse reaction. Physicians and patients should be alert to metformin-induced hepatotoxicity.

## Introduction

Metformin, a biguanide drug, is an insulin-sensitizing agent with potent antihyperglycemic properties. Metformin is currently the initial drug of choice for the treatment of type 2 diabetes mellitus (T2DM) because it reduces the risk of cardiovascular events and death in people who are overweight or obese (Holman et al., 2008).

Metformin has good safety and tolerability, and it does not increase the risk of hypoglycemia when used alone. The most common side effects of metformin are gastrointestinal reactions such as diarrhea, nausea, and/or abdominal discomfort. They are usually mild, transient, and dose-related but may occur in up to 50% of patients taking the drug (Bouchoucha et al., 2011). Lactic acidosis and vitamin B12 deficiency are additional potential side effects of metformin (Misbin et al., 1998; Out et al., 2018). Hepatotoxicity secondary to metformin is rare. Knowledge of metformin-induced hepatotoxicity is based on case reports. The clinical features and prognosis of metformin-induced hepatotoxicity are unknown. Furthermore, The diagnosis of metformininduced liver injury is difficult because of the heterogeneity of clinical presentations and the absence of established criteria. The purpose of this study was to explore the characteristics of metformin-induced hepatotoxicity and to provide reference for the diagnosis, treatment and prognosis of metformin-induced hepatotoxicity.

## Materials and methods

### Search strategy

We searched original studies, clinical reports, case reports, and reviews published in Wanfang, CNKI, VIP,PubMed, EMBASE, Web of Science, the Cochrane Library and Medline before February 2022. No language restrictions were applied. The MeSH terms and keywords were the following: metformin, hypoglycemic agents, hepatitis, hepatotoxicity, jaundice, liver injury, pruritus, cholangitis, and cholestasis. We conducted an initial assessment of the titles and abstracts of the papers and then read the full text of all potentially eligible papers. Only papers that met the following inclusion criteria were included: 1) the research subjects were humans; 2) the papers were published online; and 3) the case report included a detailed medical history, laboratory tests, treatment and prognosis.

### Data extraction

According to a self-designed table, we extracted country, age, sex, disease history, concomitant medications, indication, dose, onset time, clinical manifestations, laboratory tests, imaging studies, biopsy, treatment, and prognosis. Laboratory tests included alanine transaminase (ALT), aspartate transaminase (AST), γ-glutamyl transferase (GGT), total bilirubin (TBIL), direct bilirubin (DBIL), alkaline phosphatase (ALP), international normalized ratio (INR) and albumin (ALB).

### Relevance evaluation

The CIOMS/RUCAM (Council of International Organizations of Medical Sciences/Roussel Uclaf Method for Assessment of Causality) is used to assess drug-induced hepatotoxicity causality and states the following ratios: excluded (≤0), unlikely (1–2), possible (3–5), probable (6–8), highly probable (>8) (Danan and Benichou, 1993).

### Drug-induced liver injury severity classifications

The DILI severity classification is based on the International DILI Expert Working Group’s Severity Index (Aithal et al., 2011): 1) mild, ALT ≥5 or ALP ≥2 and TBIL<2 times the upper limit of normal (ULN); 2) moderate, ALT ≥5 or ALP ≥2 and TBIL≥2 ULN, or symptomatic hepatitis; 3) severe, ALT ≥5 or ALP ≥2 and TBIL ≥2 ULN, or symptomatic hepatitis and one of the following criteria: INR ≥1.5 or ascites and/or encephalopathy, disease duration <26 weeks, absence of underlying cirrhosis, or other organ failure due to DILI; and 4) fatal/transplantation, death or liver transplantation due to DILI.

### Pattern of liver injury

Three types of liver damage were defined: 1) hepatocellular (isolated ALT >5×ULN, or R > 5); 2) cholestatic (isolated ALP≥2×ULN, or R ≤ 2); and 3) mixed hepatocellular and cholestatic patterns (2 < R < 5). The R was defined as (measured ALT/ALT ULN)/(measured ALP/ALP ULN).^6^

### Statistical analysis

Data were analyzed descriptively. Count data are expressed as numbers and percentages, and measurement data are expressed as the median (minimum, maximum).

## Results

### Basic information

**FIGURE 1:**
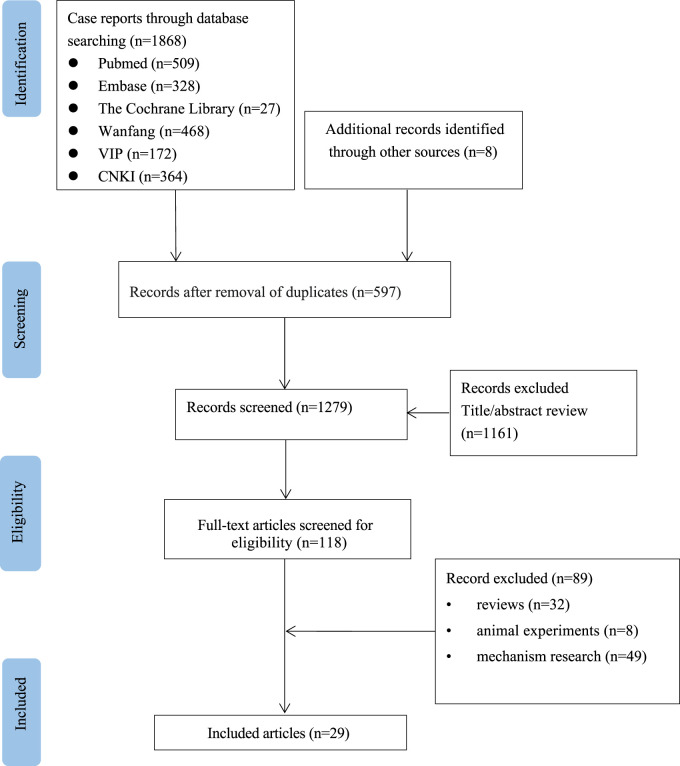
Flow chart of the study selection process for reported cases of metformin-induced hepatotoxicity.

A total of 1876 relevant studies were initially identified. After removing duplicate documents and screening the titles and abstracts, 29 studies were identified for a full-text assessment (Figure 1). The clinical characteristics of the 29 studies are summarized in Table 1. (Babich et al., 1998; Swislocki and Noth, 1998; Parikh et al., 2000; Desilets et al., 2001; Mcnear et al., 2002; Nammour et al., 2003; Deutsch et al., 2004; Barquero Romero and Pérez Miranda, 2005; Kutoh, 2005; Aksay et al., 2007; Battula et al., 2007; de la Poza Gómez et al., 2008; Biyyani et al., 2009; Cone et al., 2010; Olivera-González et al., 2010; Zhu and Xu, 2010; Hashmi, 2011; Lee et al., 2011; Mallari et al., 2011; Alston et al., 2012; Miralles-Linares et al., 2012; Ren et al., 2012; Saadi et al., 2013; Mancano, 2014; Dayanand et al., 2015; Pinto et al., 2016; Zheng, 2016; Benito et al., 2019; Chen, 2020) The median age of the 30 patients (19 males and 11 females) was 61 years (range 29–83). In addition to being used to treat type 2 diabetes, metformin was also used for weight loss in one patient (3.3%). The median time to onset of liver injury was 4 weeks (range 0.3–648). The median daily dose of metformin at the onset of liver injury was 1 g (range 0.5–2.25). Medical history information was available for 20 patients (66.7%), four of whom had a history of liver disease. Twenty-two patients (73.3%) received an average of 3.5 drugs in addition to metformin, and one patient (3.3%) had a history of drinking.

**TABLE 1:** Clinical data of 30 patients with metformin-induced hepatotoxicity.

Reference	Sex/age	Daily dose (g)	Duration	Clinical presentation	ALT	AST	TBIL	DBIL	GGT	ALP	RUCAM score	Type of injury	Resolution
Zhu and Xu, (2010)	m/48	1.5	2d	fatigue	240	104	na	na	na	na	6	H	1 m
Chen, (2020)	m/82	0.5	2 m	fatigue, bloating, loss of appetite, AD, N	158	102	1.4	0.6	68	94	9	H	2 w
Ren et al. (2012)	f/29	1.5	1y	fever, yellow urine, clay-colored stool, AD, V	782	346	1.8	na	na	na	7	H	15 d
Battula et al. (2007)	m/63	1	4 w	fever, AP, N, V	169	38	4.2	na	na	437	7	C	na
Deutsch et al. (2004)	f/67	1	6 w	fatigue, bloating, J, W	905	1152	4.8	3.5	248	121	12	C	3 m
Swislocki and Noth, (1998)	m/75	1	8 w	felt well	413	322	normal	normal	na	684	7	H	4 w
Babich et al. (1998)	f/53	2	4 w	lower extremity edema, lethargy, fatigue, diarrhea, J	651	583	14.4	na	na	500	8	M	1 m
Desilets et al. (2001)	m/64	1	2 w	fatigue, anorexia, weight loss, J	289	214	21.3	na	na	994	10	C	3 m
Nammour et al. (2003)	m/68	1.7	4 w	weight loss, pruritus, J	109	36	15.7	10	809	383	9	C	8 w
Kutoh, (2005)	f/73	0.5	3 w	fatigue, anorexia, AP, N, V, J	772	689	6.5	na	na	635	10	M	7 w
Cone et al. (2010)	m/61	1	2 w	fatigue, weight loss, N	571	623	1.8	na	325	143	9	H	2 m
de la Poza Gómez et al. (2008)	m/83	1.7	11 w	hypoxia, weight loss, J, W	47	36	2.3	na	740	586	9	C	4 m
Olivera-González et al. (2010)	f/73	2.55	2 w	anorexia, dyspnea, polyuria, polydipsia, V, W	4506	8091	1.4	na	29	95	9	H	1 m
Barquero Romero and Pérez Miranda, (2005)	f/80	1.7	8 w	loss of appetite, yellow urine, pruritus, AP, J, W	596	1198	15	12.3	442	164	8	H	3 m
Biyyani et al. (2009)	m/61	1	4 w	AP, N, V, J	169	38	4.2	1.2	na	437	9	M	4 w
Miralles-Linares et al. (2012)	m/61	1.7	6 w	J	861	290	2.9	2.4	861	622	9	M	30 d
Hashmi, (2011)	f/44	1	1 m	na	738	na	na	na	42	normal	8	H	1 m
Saadi et al. (2013)	m/78	0.85	2 w	fatigue, diarrhea, anorexia, pruritus, AP, N, V, J	1050	496	22.2	15.2	1264	1001	9	M	2 m
Zheng, (2016)	f/70	1	4 w	AD, N	1093	1152	2.2	na	na	176	8	H	10 d
Mancano, (2014)	m/41	1.5	4 w	fatigue, dark urine, clay-colored stool, J	863	419	18.1	na	2181	479	8	H	2 m
Aksay et al. (2007)	m/52	0.85	2 w	fever, N, V, W	1469	1843	2.76	1.43	na	na	9	H	10 d
Alston et al. (2012)	f/59	NA	1 m	J, N, V	85	130	5.5	na	na	293	8	C	na
Pinto et al. (2016)	f/46	2	10 y	na	92	89	0.5	0.1	na	483	8	C	na
Dayanand et al. (2015)	m/56	NA	1 m	AD, J	4701	4422	20.7	17	na	192	7	H	na
Dayanand et al. (2015)	m/61	NA	2 m	pruritus, J	1269	916	25.6	20	na	916	5	M	na
Lee et al. (2011)	m/35	NA	3 w	malaise, desquamation, AP, N, V	NA	NA	NA	na	na	NA	6	M	na
Mallari et al. (2011)	m/48	1	2 w	fatigue, malaise, AP	3165	1833	3.3	na	na	208	8	H	3 w
Parikh et al. (2000)	m/54	2	2w	fatigue, anorexia, clay colored stool, N	4ULN	4ULN	4 ULN	na	na	5 ULN	8	C	6 w
Mcnear et al. (2002)	m/55	1	few weeks	J	NA	NA	15.8	7.7	na	na	7	C	4 m
Benito et al. (2019)	f/65	NA	6 w	dark urine, AP	1414	NA	NA	na	na	na	9	H	na

Abbreviations: F, female; M, male; AD, abdominal discomfort; AP, abdominal pain; N, nausea; V, vomiting; J, jaundice; W, weakness; ALT, alanine transaminase; AST, aspartate transaminase; GGT, γ-glutamyl transferase; TBIL, total bilirubin; DBIL, direct bilirubin; ALP, alkaline phosphatase; ULN, upper limit of normal; H, Hepatocellular; C, Cholestatic; M, mixed hepatocellular and cholestatic pattern; RUCAM, roussel Uclaf Causality Assessment Method; na, not available.

### Clinical manifestations

The clinical presentation of the patients is summarized in Table 2. Twenty-eight patients (93.3%) developed symptoms at the onset of liver injury. The most common symptoms were jaundice (15 cases, 50.0%), fatigue (11 cases, 36.7%), nausea (11 cases, 36.7%), vomiting (9 cases, 30.0%), abdominal pain (8 cases, 26.7%), and anorexia (7 cases, 23.3%). Other symptoms included pruritus (4 cases, 13.3%), dark urine (4 cases, 13.3%), clay-colored stools (3 cases, 10.0%), abdominal discomfort (3 cases, 10.0%) and fever (3 cases, 10.0%). Lactic acidosis occurred in two patients (6.7%).

**TABLE 2:** Basic data of 30 patients with metformin-induced hepatotoxicity.

Parameters		Value
Sex	Male	19 (63.3%)
	Female	11 (36.7%)
Age	Year	61 (29, 83)
Country	United States	17 (56.7%)
	Spain	5 (16.7%)
	China	3 (10.0%)
	Turkey, Greece, Japanese, Israel, Saudi Arabia	1 (3.3%)
Daily dose (25)^a^	g	1 (0.5,2.25)^b^
Onset time	week	4 (0.3, 648)^b^
Indication	T2DM	29 (96.7%)
	weight loss	1 (3.3%)
HbA 1c (7)^a^	%	7.6 (7.3,11.8%)^b^
Duration	Year	0.14 (0.04, 30)^b^
Medical history (20)^a^	liver disease	4 (13.3%)
	hypertension	13 (43.3%)
	coronary heart disease	5 (16.7%)
	hyperlipidemia	5 (16.7%)
	osteoarthropathy	4 (13.3%)
	obesity	4 (13.3%)
	atrial fibrillation	2 (6.7%)
	depression	2 (6.7%)
	hypothyroidism, COPD, Crohn’s disease	1 (3.3%)
Combination therapy (22)^a^	average number of drugs	3.5
	aspirin	9 (30.0%)
	CCB	8 (26.7%)
	sulfonylureas	8 (26.7%)
	ACEI/ARB	6 (20.0%)
	statins	5 (16.7%)
	β receptor blocker	5 (16.7%)
	diuretics	5 (16.7%)
	pioglitazone	2 (6.7%)
	nateglinide, trazodone, tramadol, clomezepam hydroxychloroquine, gemfibrozil, risperidone, escitalopram, lithium carbonate, omeprazole	1 (3.3%)

Abbreviations: CCB, calcium channel blocker; ACEI/ARB, angiotensin-converting enzyme inhibitor and angiotensin receptor blocker; COPD, chronic obstructive pulmonary disease; T2DM, type 2 diabetes mellitus.

a Represents the number of patients out of 30 for whom information regarding this particular parameter was provided.

b Median (minimum, maximum).

### Laboratory tests

Laboratory test results are summarized in Table 2. The median serum ALT level was 694.5 U/L (range 47–4701), and the median serum AST level was 382.5 U/L (range 36–8091). The median TBIL level in 27 patients was 4.5 mg/dl (range 0.5–25.6), and the median DBIL level in 14 patients was 3.5 mg/dl (range 0.1–20). The median GGT level in 11 patients was 442 U/L (29–1264), and the median ALP level in 24 patients was 437 U/L (range 94–1001).

### Imaging examination

Liver imaging examination results are summarized in Table 3. Abdominal imaging was performed in 26 patients. Imaging findings were normal in 15 patients. Ultrasound in 10 patients showed hepatosteatosis (6 cases, 23.1%), gallbladder wall thickening (3 cases, 11.5%), and gallstones (1 case, 3.8%). Computed tomography (CT) in two patients showed pancreatitis (1 case, 3.8%) and hepatomegaly (1 case, 3.8%).

**TABLE 3:** Clinical manifestations and laboratory tests of 30 patients with metformin-induced hepatotoxicity.

Parameters		Value
clinical manifestations (28)^a^	jaundice	15 (50.0%)
	fatigue	11 (36.7%)
	nausea	11 (36.7%)
	vomiting	9 (30.0%)
	abdominal pain	8 (26.7%)
	anorexia	7 (23.3%)
	weakness	5 (16.7%)
	weight loss	4 (13.3%)
	pruritus	4 (13.3%)
	dark urine	4 (13.3%)
	clay colored stool	3 (10.0%)
	abdominal discomfort	3 (10.0%)
	fever	3 (10.0%)
	bloating	2 (6.7%)
	malaise	2 (6.7%)
	diarrhea	2 (6.7%)
	lactic acidosis	2 (6.7%)
	desquamation, dyspnea, polyuria, polydipsia, lower extremity edema, lethargy, hypoxia	1 (3.3%)
ALT (28)^a^	U/L	694.5 (47, 4701)^b^
AST (26)^a^	U/L	382.5 (36, 8091)^b^
TBIL (27)^a^	mg/dl	4.5 (0.5, 25.6)^b^
DBIL (14)^a^	mg/dl	3.5 (0.1, 20)^b^
ALP (24)^a^	U/L	437 (94, 1001)^b^
GGT (11)^a^	U/L	442 (29, 1264)^b^
ALB (7)^a^	g/L	39 (30, 44)^b^
Imaging examination (26)^a^	ultrasound	
	hepatosteatosis	6 (23.1%)
	thickening of the gallbladder wall	3 (11.5%)
	gallstones	1 (3.8%)
	CT	
	pancreatitis	1 (3.8%)
	hepatomegaly	1 (3.8%)
	normal	15 (57.7%)
Liver biopsy (13)^a^	portal inflammation	8 (61.5%)
	cholestatic hepatitis	5 (38.5%)
	parenchymal inflammation	5 (38.5%)
	bile duct inflammation with epithelial destruction and compensatory bile duct proliferation	3 (23.1%)
	fibrosis	2 (15.4%)
	chronic hepatitis, lymphocytic vasculitis, steatosis, severe hepatitis, pericentral necrosis	1 (7.7%)

Abbreviations: ALP, alkaline phosphatase; ALT, alanine transaminase; AST, aspartate aminotransferase; TBIL, total bilirubin; DBIL, direct bilirubin; GGT, γ-glutamyl transferase; ALB, albumin; CT, computed tomography; NA, not available.

a Represents the number of patients out of 30 for whom information regarding this particular parameter was provided.

b Median (minimum, maximum).

### Liver biopsy

Liver biopsy results are summarized in Table 3. Liver biopsies were performed in 13 patients, mainly showing portal inflammation (8 cases, 61.5%), cholestatic hepatitis (5 cases, 38.5%), parenchymal inflammation (5 cases, 38.5%) and bile duct inflammation with epithelial destruction and compensatory bile duct proliferation (3 cases, 23.1%).

### Treatments and outcomes

Metformin was discontinued immediately in all 30 patients (100%), one patient (3.3%) received the compound glycyrrhizin and a traditional Chinese medicine injection, two patients (6.7%) underwent cholecystectomy, and one patient (3.3%) underwent hemodialysis (Table 4). Liver function returned to normal levels in 30 patients (100%) at a median time of 6 weeks (range 2–16). Two patients (6.7%) had persistently high levels of ALP. Four patients (13.3%) were rechallenged with metformin, two of whom had relapsed hepatotoxicity.

**TABLE 4:** Treatment and prognosis of 30 cases of metformin-induced hepatotoxicity.

Parameters		Value
Treatment	Discounted	30 (100.0%)
	Rechanllenge	4 (13.3%)
	liver protection treatment	1 (3.3%)
	Cholecystectomy	2 (6.7%)
	Hemodialysis	1 (3.3%)
Prognosis	recover	30 (100.0%)
Resolution (24)^a^	week	6 (2, 16)^b^
RUCAM score	probable	16 (53.3%)
	highly probable	13 (43.3%)
	possible	1 (3.3%)
Pattern of liver injury	hepatocellular	14 (46.7%)
	cholestatic	9 (30.0%)
	mixed hepatocellular and cholestatic pattern	7 (23.3%)
Severity classifications	mild	5 (16.7%)
	moderate	18 (60.0%)
	severe	3 (10.0%)
	na	4 (13.3%)

Abbreviations: RUCAM, roussel Uclaf Causality Assessment Method; na, not available.

a Represents the number of patients out of 30 for whom information regarding this particular parameter was provided.

b Median (minimum, maximum).

### Causality assessment and pattern of liver injury

According to the CIOMS/RUCAM score, 16 patients (53.3%) had probable hepatotoxicity related to metformin, 13 (43.3%) had highly probable hepatotoxicity, and one patient (3.3%) had possible hepatotoxicity. Of the 30 patients, 14 (46.7%) presented with hepatocellular, 9 (30.0%) presented with cholestatic, and 7 (23.3%) presented with mixed hepatocellular and cholestatic patterns.

## Discussion

The hypoglycemic effect of metformin is mainly mediated by reducing hepatic glucose production by inhibiting gluconeogenesis and increasing glucose uptake in skeletal muscle and adipocytes. The maximum approved total daily dose of metformin for the treatment of diabetes is 2.5 g (Natali and Ferrannini, 2006). The absolute oral bioavailability of metformin is 40%–60%, and it is rapidly distributed after absorption in the small intestine and is not bound to plasma proteins. (Scheen, 1996) Hepatic uptake of metformin is mainly mediated by OCT1 (SLC22A1), and OCT3 (SLC22A3) on the hepatocyte membrane (Shu et al., 2008; Chen et al., 2015). Metformin is not metabolized by the liver and has a half-life of approximately 5 h, and 80% of the dose is excreted in the urine via the kidneys (Shu et al., 2008; Chen et al., 2015). This elimination is prolonged in patients with renal insufficiency. Epidemiological studies have shown ethnic and geographical differences in the metformin response. (Williams et al., 2014). For example, African Americans seem to have a better glycemic response to metformin than European Americans (Williams et al., 2014). Genetic and environmental factors influence individual differences in metformin adverse effects and treatment responses (Florez, 2017). Metformin-induced hepatotoxicity was seen in 20% of Asians in our analysis, with the remainder being more common in North America. More prospective studies are needed to confirm whether there are ethnic differences in metformin-induced hepatotoxicity.

The exact incidence of metformin-induced hepatotoxicity is not known, but the medication label states that liver injury is very rare (<0.01%). The type of metformin-induced hepatotoxicity is not specific and can result in hepatocellular, cholestasis, or mixed hepatocellular liver injury. The latency of metformin-induced hepatotoxicity varied widely, from 10 days to 10 years after administration. There may be differences in the susceptibility of patients of different ethnic groups to drug-induced liver injury (DILI), while metformin-induced hepatotoxicity has no obvious regionality. Advanced age may be an important susceptibility factor for DIL1 (Shu et al., 2008; Chen et al., 2015). Metformin-induced hepatotoxicity occurs mainly in diabetic patients over 50 years of age. Therefore, it is important for these patients to undergo frequent monitoring of changes in liver function during metformin use. Although sex does not appear to be a risk factor for DILI in general, it has been noted that women may show a higher susceptibility to certain drugs, such as minocycline and methyldopa (deLemos et al., 2014). In contrast, metformin-induced hepatotoxicity seems to be more common among male patients. Diabetes mellitus does not seem to increase the risk for DILI in general. It is unclear whether nonalcoholic fatty liver disease (NAFLD) and obesity increase the risk of DILI. In an alcoholic patient, liver function returned to normal after discontinuation of metformin, which ruled out an effect of alcohol on the liver (Shu et al., 2008; Chen et al., 2015).

Patients with T2DM, especially older adults, often use multiple medications due to comorbidities and complications (Zaman Huri and Fun Wee, 2013). It was brought to our attention that patients received an average of 3.5 medications other than metformin. Accompanying treatment drugs such as sulfonylureas, gemfibrozil, and statins have been reported to cause hepatotoxicity (May et al., 2002; Domínguez Tordera et al., 2011). These concomitant drugs were used before the addition of metformin, which could rule out the possibility of hepatotoxicity based on time correlation and recovery of liver function after metformin discontinuation. Nevertheless, it cannot be excluded that multidrug combination therapy contributes to metformin-induced hepatotoxicity. Data from the Swedish Prescription Drug Registry including more than 600,000 elderly (≥75 years old) patients revealed that the number of drugs was significantly associated with the occurrence of drug‒drug interactions (DDIs) (Johnell and Klarin, 2007). Metformin often interacts with a variety of drugs that may affect plasma concentrations of metformin. However, the effect of elevated plasma concentrations of metformin on liver injury is unclear.

Most of the patients with metformin-induced hepatotoxicity appeared acutely, and only serum ALT, AST, ALP, GGT, and other liver biochemical indices increased to varying degrees. Some patients may experience jaundice, fatigue, and gastrointestinal symptoms such as abdominal pain, nausea, vomiting, loss of appetite, and epigastric discomfort. Those with obvious jaundice may have yellow skin and sclera, dark urine, pale stool and pruritus. Liver biopsy demonstrated a mixed inflammatory infiltrate of the portal vein, characterized by lymphocytes, neutrophils, and numerous eosinophils. In contrast, acute inflammatory cells infiltrate the bile ducts with epithelial destruction and compensatory bile duct proliferation.

The pathophysiological mechanism of metformin-induced hepatotoxicity remains unclear. Metformin is not hepatically metabolized and is generally not considered to be toxic to the liver. Possible mechanisms of injury are direct, idiosyncratic, or a drug‒drug interaction leading to acute liver injury in this susceptible individual. Some patients with fever and liver biopsy showed eosinophilic infiltration, supporting this point of view. Due to the direct blood supply from the portal vein, the concentrations of metformin in the liver may be much higher than those in the systemic circulation and other organs. Although metformin is concentrated in the liver, there is no evidence of dose-dependent hepatotoxicity (Wilcock and Bailey, 1994). However, the effect of elevated plasma concentrations of metformin on liver injury is unclear. The relationship between metformin-induced hepatotoxicity and gene polymorphisms still needs further research.

Timely discontinuation of suspected liver injury drugs is the most important treatment measure for DILI, and rechallenging suspected or similar drugs should be avoided as much as possible. Appropriate drug therapy is selected according to the clinical type of DILI (May et al., 2002; Domínguez Tordera et al., 2011). However, most patients with DILI will spontaneously recover without any treatment or specific measures after discontinuation of the suspected drug. A small number of patients develop chronic liver disease, and very few develop acute liver failure or even die (May et al., 2002; Domínguez Tordera et al., 2011). In our study, all patients had normal liver function within 4 months after discontinuation of metformin without any intervention. Persistently high levels of ALP in two patients were thought to be associated with long-term cholestatic effects (Nammour et al., 2003; Biyyani et al., 2009). Cholecystectomy may be required for metformin-induced cholangiohepatitis (Battula et al., 2007; Biyyani et al., 2009). One patient with acute kidney injury and lactic acidosis secondary to acute liver failure underwent hemodialysis (Battula et al., 2007; Biyyani et al., 2009). The effects of readministration of metformin remains uncertain, as some patients do not experience recurrent hepatotoxicity after metformin rechallenge (Swislocki and Noth, 1998; Zhu and Xu, 2010).

## Conclusion

Metformin-induced liver injury is rare and easily overlooked due to its insidious onset. Given the increasing prevalence of T2DM and the widespread use of metformin, clinicians should be alert to metformin-induced hepatotoxicity, a rare but potentially serious adverse effect. It should be reminded that when the patients have symptoms such as jaundice, fatigue, anorexia, pruritus, and dark urine during the medication, they should seek medical attention in time for necessary examinations, especially about 1 month after starting the medication.

## Data Availability

The original contributions presented in the study are included in the article/supplementary material, further inquiries can be directed to the corresponding author
